# Supplementary figures and images for: Effects of swimming before and during pregnancy on placental angiogenesis and perinatal outcome in high-fat diet-fed mice
Source: PeerJ. 2023 Feb 20;11:e14562. doi: 10.7717/peerj.14562 (PMC9948747; doi:10.7717/peerj.14562)

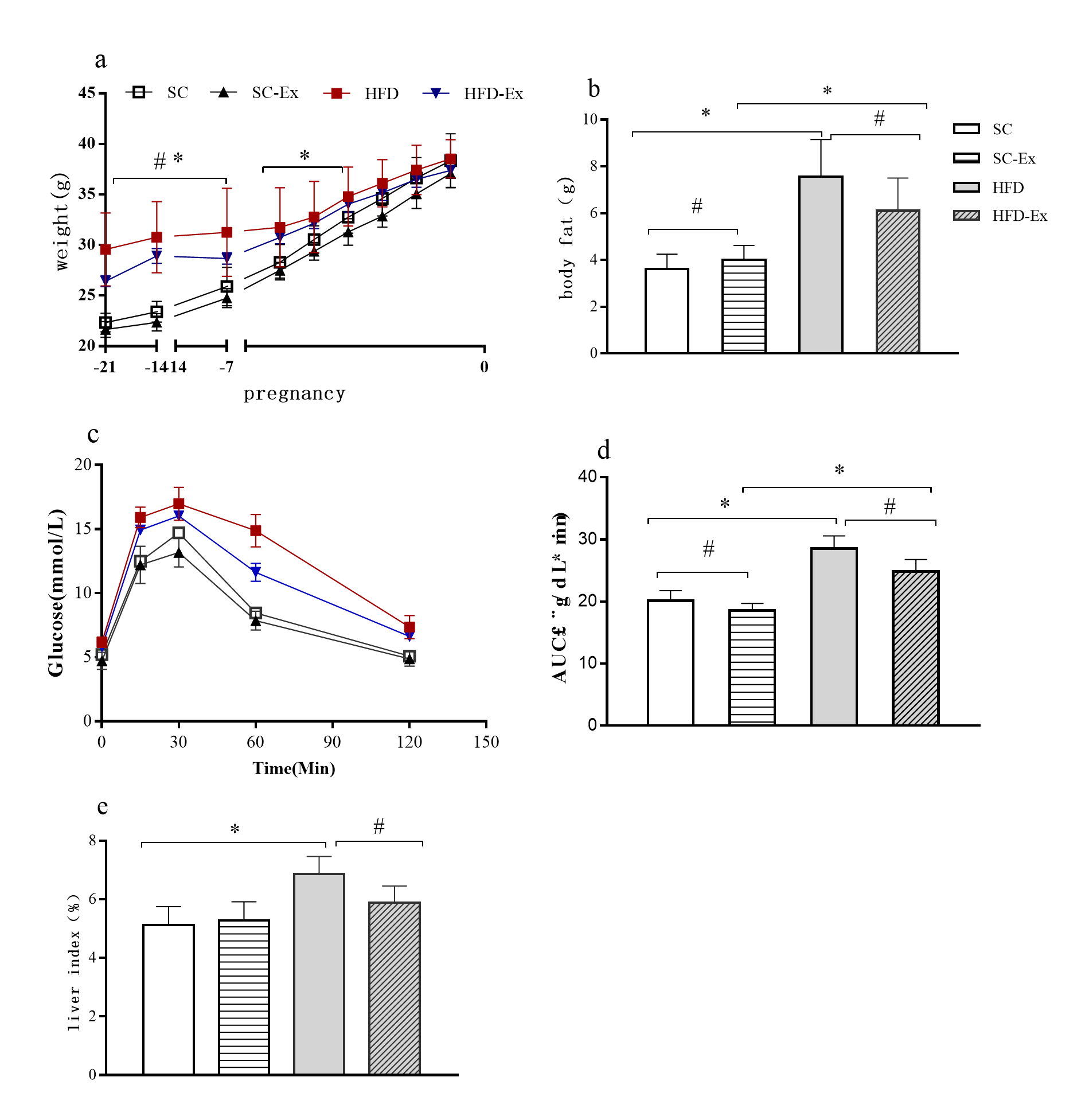

Supplement: Supplemental Information 2 — (A) Body weight. (B) Body fat. (C) Glucose tolerance. (D) Area under the receiver operating characteristic curve (AUC). (E) Liver index. The body weight was measured on the day of fertilization (F1), monitored once a week in the following two weeks, and monitored daily in the last week. [file peerj-11-14562-s002.tif]
